# Predictors of pneumococcal carriage and the effect of the 13-valent pneumococcal conjugate vaccination in the Western Australian Aboriginal population

**DOI:** 10.1186/s41479-017-0038-x

**Published:** 2017-09-25

**Authors:** Deirdre A. Collins, Anke Hoskins, Thomas Snelling, Kalpani Senasinghe, Jacinta Bowman, Natalie A. Stemberger, Amanda J. Leach, Deborah Lehmann

**Affiliations:** 1 School of Medical and Health Sciences, Edith Cowan University, Perth, WA Australia; 2 Wesfarmers Centre of Vaccines & Infectious Diseases, Telethon Kids Institute, University of Western Australia, Perth, WA Australia; 3 Division of Microbiology & Infectious Diseases, PathWest Laboratory Medicine WA, Perth, WA Australia; 4 Menzies School of Health Research, Charles Darwin University, Darwin, Northern Territory Australia

**Keywords:** *Streptococcus pneumoniae*, Colonization, Pneumococcal disease, Aboriginal, Vaccination, Australia, PCV13

## Abstract

**Background:**

The 7-valent pneumococcal conjugate vaccine (PCV7) was introduced to prevent invasive pneumococcal disease (IPD) in Western Australian (WA) Aboriginal people in 2001. PCV13 replaced PCV7 in July 2011, covering six additional pneumococcal serotypes; however, IPD rates remained high in Aboriginal people in WA. Upper respiratory tract pneumococcal carriage can precede IPD, and PCVs alter serotype distribution.

**Methods:**

To assess the impact of PCV13 introduction, identify emerging serotypes, and assess risk factors for carriage, nasopharyngeal swabs and information on demographic characteristics, health, medication and living conditions from Aboriginal children and adults across WA from August 2008 to November 2014 were collected. Bacteria were cultured using selective media and pneumococcal isolates were serotyped by Quellung reaction. Risk factors were analysed by multivariable logistic regression.

**Results:**

One thousand five hundred swabs pre- and 1385 swabs post-PCV13 introduction were collected. Pneumococcal carriage was detected in 66.8% of children <5 years old and 53.2% of 5–14 year-olds post-PCV13, compared with pre-PCV13 prevalence of 72.2% and 49.4%, respectively. The prevalence of PCV13-non-PCV7 serotypes decreased in children <5 years old from 13.5% pre-PCV13 to 5.8% post-PCV13 (*p* < 0.01), and from 8.4% to 6.1% in children 5–14 years old (*p* > 0.05). The most common serotypes post-PCV13 were 11A (prevalence 4.0%), 15B (3.5%), 16F (3.5%), and 19F (3.2%).

Risk of detection of pneumococcal carriage increased until age 12 months (odds ratio [OR] 4.19, 95% confidence interval [CI] 2.39–7.33), with nasal discharge (OR 2.49 [95% CI 2.00–3.09]), residence in a remote community (OR 2.21 [95% CI 1.67–2.92]) and household crowding (OR 1.36 [95% CI 1.11–1.67]). Recent antibiotic use was negatively associated with pneumococcal carriage (OR 0.48 [95% CI 0.33–0.69]). Complete resistance to penicillin was present among isolates of serotypes 19A (6.0%), 19F (2.3%) and non-serotypeable isolates (1.9%). Serotype 23F and newly emerged serotype 7B isolates showed high rates of resistance to cotrimoxazole, erythromycin and tetracycline (86.9%, 86.9%, 82.0%, respectively for 23F, 100.0%, 100.0% and 93.3% for 7B).

**Conclusion:**

Since PCV13 replaced PCV7, carriage of PCV13-non-PCV7 serotypes decreased significantly among children <5 years old, those most likely to have received PCV13, and to a lesser extent in older people. Known risk factors for carriage including crowding and young age remain in the Aboriginal population.

## Background

*Streptococcus pneumoniae* is estimated to cause up to a million deaths from invasive pneumococcal disease (IPD) per year, worldwide [1]. While the greatest burden of IPD is concentrated in resource-poor countries, Australian Aboriginal people in central and western regions of Australia experience an almost five-fold greater incidence of IPD than non-Aboriginal Australians [2]. In addition to high rates of IPD, Aboriginal children also experience a high burden of other manifestations of pneumococcal disease, including pneumonia and otitis media (OM) [3].

Vaccination to prevent IPD is effective against targeted serotypes, but limited by the existence of at least 93 serotypes of *S. pneumoniae*, each differentiated by its polysaccharide capsule. The 7-valent pneumococcal conjugate vaccine (PCV7, covering serotypes 4, 6B, 9V, 14, 18C, 19F and 23F) was introduced for Australian Aboriginal children at 2, 4 and 6 months old in 2001 with a booster dose of 23-valent pneumococcal polysaccharide vaccine (23vPPV, covering PCV7 serotypes and 1, 2, 3, 5, 7F, 8, 9 N, 10A, 11A, 12F, 15B, 17F, 19A, 20, 22F, and 23F) scheduled at 18 months old. A catch-up schedule was in place for children <2 years old and for children <5 years old with predisposing medical conditions. All Australian children became eligible in 2005 for 3 primary doses of PCV7 with no scheduled booster. Following the introduction of PCV7, IPD rates initially fell in the Aboriginal population in Western Australia (WA) but subsequently increased in adults [2]. The observed increase was accompanied by a rise in IPD caused by non-PCV7 serotypes [4, 5].

On 1 July 2011, PCV7 was recalled and immediately replaced with PCV13 (covering the six additional serotypes 1, 3, 5, 6A, 7F and 19A). For Aboriginal children, a fourth dose of PCV13 at 18 months old replaced the 23vPPV booster over a transition period from September 2011 to October 2012 [6]. Following PCV13 introduction in Australia, IPD rates appeared to decrease; however, rates in IPD caused by non-PCV13 serotypes increased [4, 7].

Nasopharyngeal carriage of *S. pneumoniae* is generally asymptomatic but is an important precursor to pneumococcal disease. Pneumococcal carriage studies have been used to monitor the prevalence and distribution of *S. pneumoniae* serotypes in WA, and for surveillance of antibiotic resistant strains. Despite introduction of the PCV program, pneumococcal carriage rates remain high among Aboriginal people [8, 9], and have been observed to be higher in Aboriginal children than in non-Aboriginal children [10]. In WA, carriage rates were 71.9% and 34.6% among Aboriginal children <5 years old and people ≥5 years old, respectively, after introduction of PCV7 [9]. Most pneumococcal carriage serotypes in WA in the PCV7 era were non-PCV7 serotypes, the most common being 19A, 16F and 6C [9].

Given that the PCV vaccination program does not appear to have reduced overall IPD rates in the Aboriginal population in age groups other than children <5 years [4], it is important to identify risk factors for carriage of *S. pneumoniae* to help identify further opportunities for prevention of pneumococcal disease. Previously, household crowding and exposure to environmental tobacco smoke (ETS) have been identified as risk factors [11]. The authors have monitored pneumococcal carriage in Aboriginal people living in WA since 2008. This study describes the prevalence of overall carriage and that of individual serotypes in the WA Aboriginal population before and after the introduction of PCV13, along with epidemiological risk factors for carriage.

## Methods

### Study population

Aboriginal people make up about 3% of the total WA population (total 2.2 million people); 39% live in the Perth metropolitan area, the remainder in regional towns or scattered sparsely across remote areas of WA. The WA climate varies, from tropical in the north to inland deserts and warm temperate southwest coastal regions.

### Sample and data collection and laboratory analysis

The surveillance study has been described previously [9]. In brief, nasopharyngeal swabs or nose blown samples [12] were collected opportunistically from Aboriginal people of all ages in communities across WA from August 2008 to November 2014. Participants were defined as having participated “pre-PCV13” introduction (from study start until 30 June 2011) or “post-PCV13” (from 1 July 2011 onwards). Demographic, environmental and health data including details on smoking behaviour and exposure, numbers of co-resident household members, recent medication and illness were collected by completing a questionnaire during face-to-face interviews. Swabs were cultured on selective media. *S. pneumoniae* isolates were confirmed by optochin susceptibility. Two pneumococcal isolates (or more, if morphologically distinct) per positive culture were subcultured and serotyped by the Quellung reaction using antisera from the Statens Serum Institut, Denmark [13]. Isolates that could not be serotyped by Quellung reaction were referred to as “non-serotypeable”. Antimicrobial susceptibilities were tested by disc diffusion. E-test (bioMérieux Diagnostics, France) was performed where reduced antimicrobial susceptibility was determined by disc diffusion. Resistance to antimicrobials was classified according to the Clinical and Laboratory Standards Institute guidelines [14]. Breakpoints for intermediate and complete resistance to penicillin (collectively termed “non-susceptibility”) were 4 μg/mL and 8 μg/mL, respectively.

The remoteness (metropolitan, regional or remote) of communities was classified using the Australian Statistical Geography Standard Remoteness Structure [15]. Household crowding was defined as ≥5 people sharing accommodation on the night prior to sample collection. Co-residence with a child was defined as sharing with at least one child <5 years old on the previous night. Respiratory symptoms were defined as any cough, sore throat, or blocked or runny nose as reported by the participant or guardian. Environmental tobacco smoke (ETS) exposure was considered present if it was reported that any co-resident was a smoker. The immunization status of children was determined from the Australian Childhood Immunization Register (ACIR), accessed using the participant’s name and date of birth. Children were classified as vaccinated with PCV7 or PCV13 if they had received at least two doses of the relevant vaccine at least 2 weeks prior to specimen collection.

### Statistical analysis

All descriptive and statistical analyses were performed using SPSS v22.0 (IBM Corp., Armonk, New York, United States of America [USA]) and Stata 11 (Stata Corporation, College Station, Texas, USA). Results of culture and serotyping were aggregated according to serotype to calculate the prevalence of individual serotypes among all study participants. Differences in crude proportions were compared using χ^2^ test; statistical significance was considered if *p* < 0.05. A multivariable logistic regression model was used to identify independent risk factors for carriage of serotypeable pneumococci. Variables were included, if identified previously in the literature as risk factors for carriage, in a backwards stepwise model, eliminating variables using a cut-off value of *p* > 0.05. Vaccination status was excluded from the risk factor analysis due to poor recovery of vaccination records and low number of PCV13-vaccinated children (see Results section). The influence of age on carriage risk was modelled using a spline function with knots at age 1 year and at 20 years, based on previous findings of a peak in carriage at 12 months of age in this particular population [9]. The influence of calendar time was modelled as a linear continuous variable, with the influence of the PCV13 program on carriage explored using a simple marginal spline function with a single knot corresponding to 1 July 2011, the date of PCV13 introduction.

### Ethical approval

Approval was granted by the Princess Margaret Hospital for Children Ethics Committee, the Western Australian Country Health Service Board Research Ethics Committee and the Western Australian Aboriginal Health Ethics Committee. Approval to approach communities in the Kimberley region was granted by the Kimberley Aboriginal Health Planning Forum. Consent was sought from adult participants and from the parents or guardians of child participants.

## Results

### Study population

In total 2885 nasopharyngeal samples (2824 nasopharyngeal swabs; 60 nose blown samples; 1 unknown) were collected: 1500 before the introduction of PCV13 on 1 July 2011, and 1385 afterwards. Most participants reported being healthy on the day of participation, while 31.5% had visible nasal discharge and 8.4% reported respiratory symptoms (Table 1). Most samples were collected among remote residents (78.2%). Crowding (61.7%) and co-residence with children (73.5%) were common across all age groups. Any ETS exposure was reported by 65.3% of participants; 22.9% reported exposure to indoor ETS, and 55.6% of adult respondents reported smoking themselves. Recent antibiotic use was reported by 7.6% of participants. Immunization status could be determined for only 628 (59.1%) participants <5 years old and 488 (51.5%) participants 5–14 years old. Among PCV7 age-eligible children whose immunization status was available, 504 (89.4%) of those <5 years old and 302 (74.6%) of those aged 5–14 years were vaccinated with PCV7 (Table 1). Among children eligible for PCV13 whose immunization status was known, 37 (59.7%) were vaccinated with at least two doses of PCV13 and 64 (98.5%) had received at least one dose. A combination of PCV7 and PCV13 (at least one dose of each) was received by 66 (6.8%) children.

**Table 1:** Descriptive characteristics of Aboriginal people in Western Australia enrolled before and after PCV13 introduction (1 July 2011), by age group (August 2008–November 2014)

	*n* (%)						
	<5 years		5–14 years		≥15 years		
	*pre-PCV13*	*post-PCV13*	*pre-PCV13*	*post-PCV13*	*pre-PCV13*	*post-PCV13*	Total
*S. pneumoniae* carriage							
Any serotype	406 (72.2)	334 (66.8)	235 (49.4)	251 (53.2)	90 (19.5)	41 (9.9)**	1357 (47.0)
PCV7 serotype	59 (10.5)	32 (6.4)*	35 (7.4)	28 (5.9)	9 (1.9)	5 (1.2)	168 (5.8)
PCV13-nonPCV7 serotype	76 (13.5)	29 (5.8)**	40 (8.4)	29 (6.1)	10 (2.2)	4 (1.0)	188 (6.5)
PCV13 serotype	133 (23.7)^a^	60 (12.0)^b^**	74 (15.5)^b^	57 (12.1)	19 (4.1)	9 (2.2)	352 (12.2)
Male	307 (54.6)	252 (50.6)	249 (52.3)	223 (47.2)	117 (25.3)	74 (17.9)	1222 (42.4)
Respiratory symptoms	65 (13.6)	66 (13.2)	14 (3.0)	20 (4.2)	26 (5.7)	44 (10.7)	235 (8.4)
Nasal discharge	294 (60.2)	234 (46.8)**	170 (36.2)	111 (23.5)**	31 (6.8)	43 (10.4)	883 (31.5)
Remoteness index							
Metropolitan	68 (12.1)	71 (14.2)	62 (13.0)	76 (16.1)	26 (5.6)	63 (15.3)	366 (12.7)
Regional	43 (7.7)	73 (14.6)	20 (4.2)	40 (8.5)	29 (6.3)	57 (13.8)	262 (9.1)
Remote	451 (80.2)	356 (71.2)	394 (82.8)	356 (75.4)	407 (88.1)	293 (70.9)	2257 (78.2)
≥5 people sharing (crowding)	308 (65.7)	280 (57.1)**	291 (66.1)	294 (66.4)	282 (66.4)	194 (47.8)**	1649 (61.7)
Sharing with ≥1 child <5 years	368 (78.6)	326 (66.5)**	336 (76.4)	303 (69.0)*	353 (83.6)	272 (67.3)**	1958 (73.5)
Antibiotic use in previous 2 weeks	46 (8.5)	60 (12.3)	13 (2.7)	35 (7.7)**	23 (5.0)	39 (9.5)*	216 (7.6)
Indoor environmental tobacco smoke exposure	103 (22.0)	103 (21.2)	91 (20.6)	96 (21.7)	131 (29.6)	91 (22.5)*	615 (22.9)
Any environmental tobacco smoke exposure	325 (67.6)	340 (69.7)	272 (59.4)	287 (64.3)	297 (66.6)	260 (63.9)	1781 (65.3)
Smoker	N/A	N/A	N/A	N/A	237 (59.1)	182 (52.1)	419 (55.6)
Season							
Summer	43 (7.7)	23 (4.6)	51 (10.7)	20 (4.2)	18 (3.9)	22 (5.3)	177 (6.1)
Autumn	108 (19.2)	94 (18.8)	103 (21.6)	82 (17.4)	91 (19.7)	93 (22.5)	571 (19.8)
Winter	275 (48.9)	242 (48.4)	204 (42.9)	183 (38.8)	169 (36.6)	190 (46.0)	1263 (43.8)
Spring	136 (24.2)	141 (28.2)	118 (24.8)	187 (39.6)	184 (39.8)	108 (26.2)	874 (30.3)
PCV7 vaccinated	360 (88.9)	144 (90.6)	168 (69.4)	134 (82.2)	N/A	N/A	806 (83.2)
PCV13 vaccinated^c^	N/A	37 (59.7)	N/A	N/A	N/A	N/A	37 (26.2)
Vaccination status unknown	151 (26.8)	283 (56.6)	165 (34.7)	295 (62.5)	N/A	N/A	894 (44.5)
*H. influenzae*	355 (63.2)	274 (54.8)**	170 (35.7)	186 (39.4)	40 (8.7)	24 (5.8)	1049 (36.4)
*M. catarrhalis*	353 (62.8)	356 (71.2)**	193 (40.5)	175 (37.1)	60 (13.0)	30 (7.3)**	1167 (40.5)
*S. aureus*	64 (11.4)	30 (6.0)**	73 (15.3)	77 (16.3)	46 (10.0)	35 (8.5)	325 (11.3)

Denominators for proportions varied due to missing data. Proportions were compared using χ^2^

*p < 0.05, **p < 0.01

^a^Two participants carried a PCV7 and PCV13-non-PCV7 serotype simultaneously

^b^One participant carried a PCV7 and PCV13-non-PCV7 serotype simultaneously

^c^Denominator is number of participants at time of enrolment pre/post PCV13. Includes participants vaccinated with PCV7 prior to introduction of PCV13

### Carriage prevalence

**Fig. 1:**
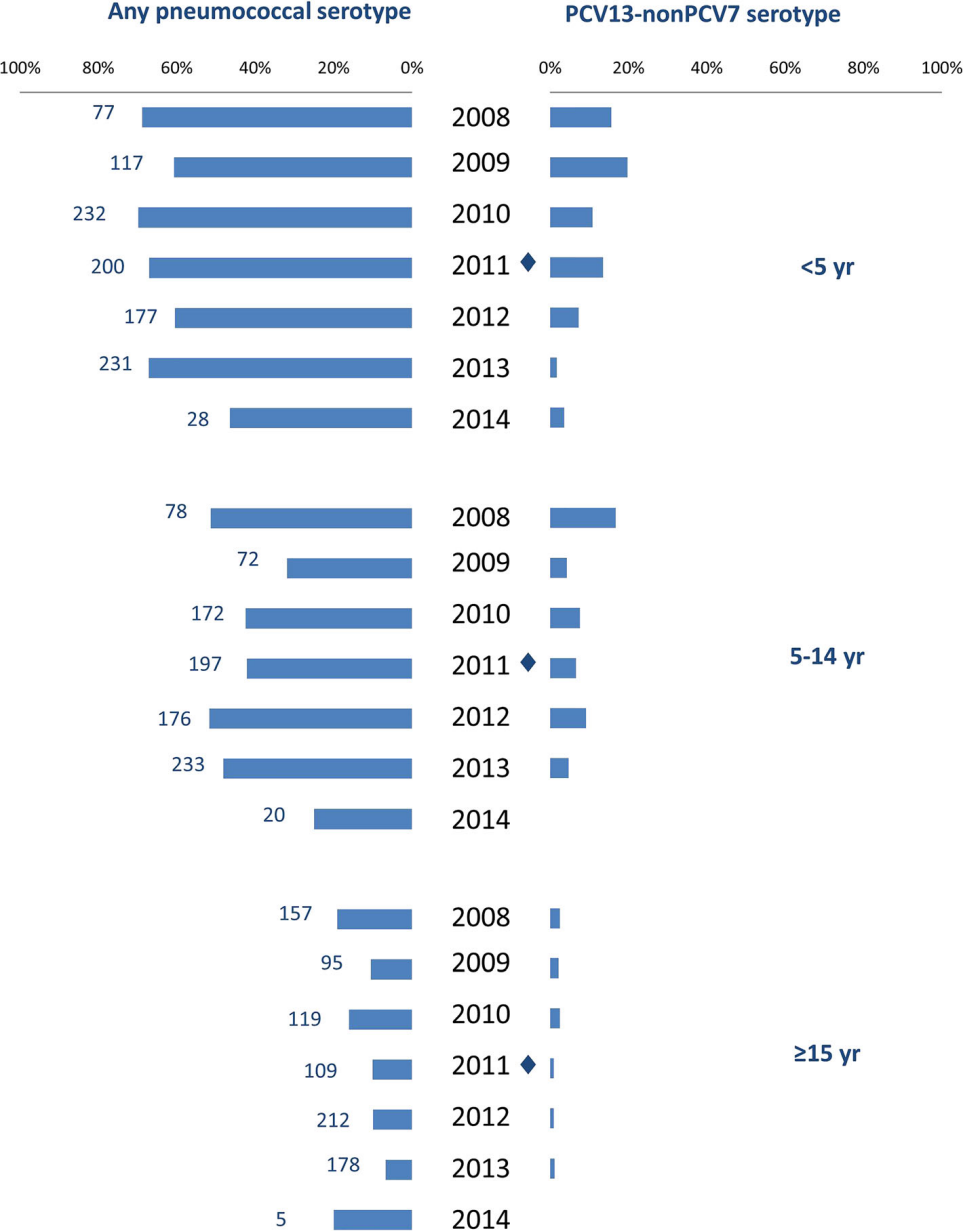
Prevalence of any serotypeable pneumococcus or PCV13-nonPCV7 serotype only by year, by age group, among Aboriginal people in Western Australia, August 2008–November 2014. Numbers by bars represent number of participants per year. ◆PCV13 was introduced on 1 July 2011

Compared to the pre-PCV13 period, carriage of any pneumococcal serotype was less common in the post-PCV13 period among adults (19.5% versus 9.9%, *p* < 0.01) and to a lesser extent among children <5 years old (72.2% versus 66.8%, *p* = 0.054) but not in children 5–14 years old (49.4% versus 53.2%, *p* = 0.24). Carriage of PCV13-non-PCV7 serotypes was less common post-PCV13 introduction among children <5 years old (13.5% versus 5.8%, *p* < 0.01; Table 1, Fig. 1). There was also a numerical reduction in carriage of vaccine serotypes in older age groups (Table 1). *S. pneumoniae* was recovered from 46.8% of nasopharyngeal swabs and 56.7% of nose-blown samples.

**Fig. 2:**
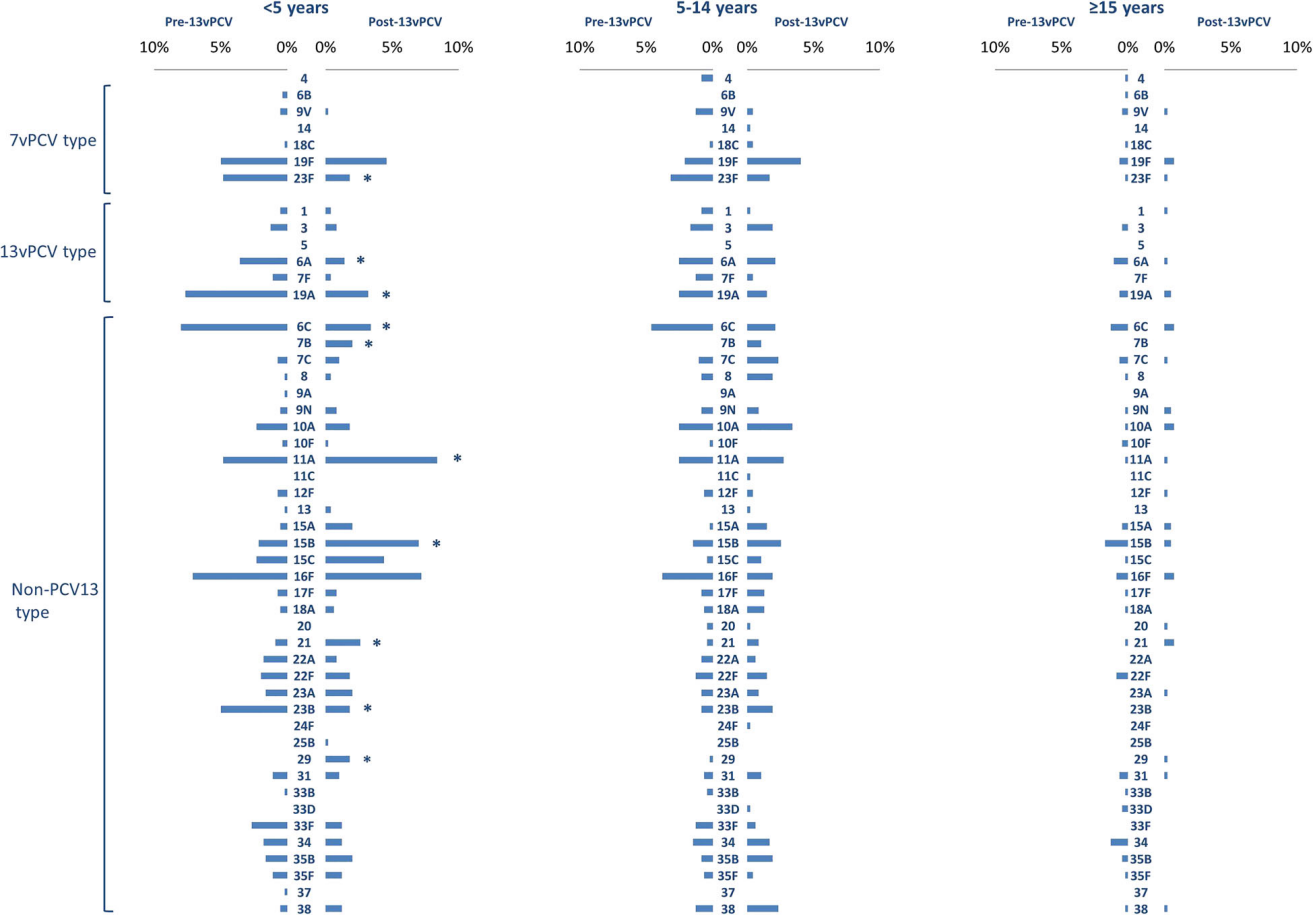
Prevalence of serotypeable isolates by age group, pre- and post-PCV13 introduction (1 July 2011), among Aboriginal people in Western Australia, August 2008 to November 2014. *p < 0.0

Culture identified 1357 pneumococcus-positive specimens (47.0%) giving 1590 distinct pneumococcal isolates, 1374 (86.4%) of which were serotypeable (Table 2). The most common of the 48 serotypes detected were 16F (prevalence 3.8%), 6C (3.6%), 11A (3.3%), 19F (3.0%), 19A (2.9%) and 15B (2.6%). Several PCV13 serotypes decreased in frequency post-PCV13 introduction in children <5 years old: PCV7 type 23F (4.8% versus 1.8%, *p* < 0.05), PCV13-non-PCV7 types 6A (3.6% versus 1.4%, *p* < 0.05) and 19A (7.7% versus 3.2%, *p* < 0.05) (Fig. 2). Reductions in PCV13 types were also observed in participants ≥5 years old, to a lesser extent than that seen in children <5 years (15.5% to 12.1% in 5–14 years, *p* = 0.12; 4.1% to 2.2% in ≥15 years group, *p* = 0.10, Table 1).

**Table 2:** Frequencies of serotypes and antibiotic resistance rates found among 1590 isolates of *Streptococcus pneumoniae* among Aboriginal people in Western Australia, August 2008–November 2014*.* PCV13 was introduced on July 1 2011

	Isolates		pen^I^ (%)		pen^R^ (%)		cotr^R^ (%)		ery^R^ (%)		tet^R^ (%)	
Serotype	Pre (*n*)	Post (*n*)	Pre	Post	Pre	Post	Pre	Post	Pre	Post	Pre	Post
16F	62	48	32.3	25.0	0.0	0.0	0.0	0.0	8.1	0.0	6.5	0.0
6C	73	30	0.0	0.0	0.0	0.0	64.4	43.3	4.1	3.3	0.0	0.0
11A	40	56	7.5	10.7	0.0	0.0	17.5	26.8	2.5	0.0	0.0	0.0
19F	41	45	90.2	75.6	0.0	4.4	0.0	4.4	0.0	6.7	0.0	4.4
19A	58	25	39.7	52.0	5.2	8.0	43.1	56.0	10.3	24.0	25.9	44.0
15B	27	49	14.8	2.0	0.0	0.0	7.4	18.4	11.1	8.2	0.0	0.0
23F	43	18	4.7	0.0	0.0	0.0	81.4	100.0	81.4	100.0	76.7	94.4
6A	37	18	2.7	0.0	0.0	0.0	8.1	11.1	59.5	50.0	2.7	0.0
10A	26	28	3.8	0.0	0.0	0.0	61.5	35.7	0.0	0.0	0.0	0.0
23B	32	18	18.8	38.9	0.0	0.0	0.0	11.1	0.0	0.0	0.0	0.0
15C	16	27	6.3	3.7	0.0	0.0	25.0	18.5	0.0	7.4	0.0	0.0
34	23	14	0.0	0.0	0.0	0.0	0.0	0.0	0.0	7.1	0.0	0.0
22F	21	16	0.0	0.0	0.0	0.0	0.0	0.0	71.4	50.0	0.0	0.0
35B	15	19	13.3	15.8	0.0	0.0	6.7	0.0	0.0	10.5	0.0	0.0
3	17	13	0.0	7.7	0.0	0.0	0.0	0.0	0.0	0.0	0.0	0.0
33F	21	9	4.8	0.0	0.0	0.0	4.8	11.1	0.0	22.2	0.0	11.1
7C	12	17	0.0	0.0	0.0	0.0	0.0	0.0	16.7	70.6	0.0	0.0
21	8	20	0.0	0.0	0.0	0.0	37.5	10.0	0.0	5.0	0.0	0.0
23A	13	15	0.0	6.7	0.0	0.0	0.0	0.0	0.0	0.0	0.0	0.0
38	10	18	0.0	0.0	0.0	0.0	0.0	0.0	0.0	0.0	0.0	22.2
15A	6	19	50.0	73.7	0.0	0.0	33.3	31.6	83.3	73.7	50.0	73.7
22A	14	7	7.1	0.0	0.0	0.0	0.0	0.0	0.0	0.0	0.0	0.0
31	12	12	0.0	0.0	0.0	0.0	0.0	0.0	16.7	0.0	0.0	0.0
17F	9	10	11.1	0.0	0.0	0.0	0.0	0.0	55.6	10.0	0.0	0.0
9 N	8	10	0.0	0.0	0.0	0.0	0.0	0.0	75.0	40.0	75.0	50.0
35F	10	8	0.0	0.0	0.0	0.0	0.0	0.0	0.0	0.0	0.0	0.0
8	6	11	0.0	0.0	0.0	0.0	0.0	0.0	0.0	0.0	0.0	0.0
7F	12	4	0.0	0.0	0.0	0.0	0.0	0.0	8.3	0.0	0.0	0.0
18A	7	9	0.0	0.0	0.0	0.0	0.0	0.0	0.0	0.0	0.0	0.0
7B	0	15	0.0	100.0	0.0	0.0	0.0	100.0	0.0	100.0	0.0	93.3
9 V	11	4	100.0	100.0	0.0	0.0	100.0	100.0	0.0	25.0	0.0	0.0
1	7	4	14.3	0.0	0.0	0.0	0.0	0.0	0.0	0.0	0.0	0.0
29	1	10	0.0	0.0	0.0	0.0	0.0	0.0	0.0	0.0	0.0	0.0
12F	7	3	0.0	0.0	0.0	0.0	0.0	0.0	0.0	0.0	0.0	0.0
10F	5	1	0.0	0.0	0.0	0.0	100.0	100.0	0.0	0.0	0.0	0.0
4	5	0	20.0	0.0	0.0	0.0	20.0	0.0	0.0	0.0	0.0	0.0
18C	3	2	0.0	0.0	0.0	0.0	100.0	100.0	0.0	0.0	0.0	0.0
13	1	3	0.0	33.3	0.0	0.0	0.0	0.0	0.0	33.3	0.0	0.0
20	2	2	0.0	0.0	0.0	0.0	0.0	0.0	0.0	0.0	0.0	0.0
33B	4	0	50.0	0.0	0.0	0.0	0.0	0.0	50.0	0.0	0.0	0.0
6B	3	0	0.0	0.0	0.0	0.0	0.0	0.0	33.3	0.0	0.0	0.0
33D	2	1	100.0	100.0	0.0	0.0	0.0	0.0	100.0	100.0	0.0	0.0
9A	1	0	0.0	0.0	0.0	0.0	0.0	0.0	0.0	0.0	0.0	0.0
11C	0	1	0.0	0.0	0.0	0.0	0.0	0.0	0.0	0.0	0.0	0.0
14	0	1	0.0	0.0	0.0	0.0	0.0	0.0	0.0	100.0	0.0	0.0
24F	0	1	0.0	0.0	0.0	0.0	0.0	0.0	0.0	0.0	0.0	0.0
25B	0	1	0.0	0.0	0.0	0.0	0.0	0.0	0.0	0.0	0.0	0.0
37	1	0	0.0	0.0	0.0	0.0	0.0	0.0	0.0	0.0	0.0	0.0
NT	126	90	76.2	63.3	0.0	0.0	46.0	38.9	34.1	23.3	9.5	11.1
TOTAL	858	732	25.5	23.4	0.3	0.5	26.1	21.3*	18.5	17.5	8.6	10.7

48 serotypes were identified among 1374 serotypeable isolates, 216 isolates were non-serotypeable (NT)

*Cotr* cotrimoxazole, *ery* erythromycin, *tet* tetracycline, *pen* penicillin; ^*R*^ resistant, ^*I*^ intermediate resistance

*p < 0.05

Carriage of more than one serotype was identified in 131 (4.5%) specimens. Non-serotypeable isolates were detected in addition to a serotypeable isolate in 81 (2.8%) specimens. The prevalence of a non-serotypeable pneumococcus in the absence of serotypeable strains was lower post-PCV13 (4.7% versus 3.9%, *p* = 0.06).

Among 36 non-PCV13 serotypes identified, the prevalence of 11A (4.8% versus 8.4%, *p* < 0.05), 15B (2.1% versus 7.0%, *p* < 0.01) and 21 (0.9% versus 2.6%, *p* < 0.05) increased significantly among children <5 years old while 7B and 29 were only detected post-PCV13 (2.0% and 1.8% respectively, both *p* < 0.05). Serotypes 6C and 23B were detected less frequently post-PCV13 introduction in children <5 years old (8.0% versus 3.4%, *p* < 0.05 and 5.0% versus 1.8%, *p* < 0.05; Fig. 2).

### Antimicrobial susceptibility

Non-susceptibility to penicillin was present in 25.2% of isolates, including in 84.9% of serotype 19F isolates. Complete resistance to penicillin was uncommon, found among isolates of serotypes 19A (6.0%), 19F (2.3%) and non-serotypeable isolates (1.9%) only (Table 2). Serotype 23F isolates showed high rates of resistance to cotrimoxazole, erythromycin and tetracycline (86.9%, 86.9% and 82.0%, respectively). Multi-resistance to three or more antibiotics was recorded in 102 serotypeable isolates (7.4%) overall; mainly serotype 15A isolates (68.0%) and serotype 23F isolates (88.5%). Isolates of serotype 7B (*n* = 15), which were only detected post-PCV13, were all resistant to cotrimoxazole and erythromycin, non-susceptible to penicillin and 93.3% were resistant to tetracycline (Table 2). Overall, susceptibility of strains to antimicrobials did not change significantly after introduction of PCV13, apart from a decrease in cotrimoxazole resistance (26.1% to 21.3%, *p* < 0.05, Table 2). Multi-resistance was present in 17.6% of non-serotypeable isolates.

### Risk factors for pneumococcal carriage

Carriage of any serotypeable pneumococcus was more common among males (OR 1.56 [95% CI 1.35–1.81]) and those who were carriers of *Haemophilus influenzae* (OR 10.29 [95% CI 8.60–12.30]) and *Moraxella catarrhalis* (OR 7.10 [95% CI 6.01–8.39]) compared with those who were not (Table 3). Pneumococcal carriage was detected more commonly among those reporting respiratory symptoms (OR 1.48 [95% CI 1.14–1.94]), in the presence of nasal discharge (OR 4.84 [95% CI 4.01–5.74]), among those living in a remote community (OR 1.86 [95% CI 1.41–2.35]) and those living in crowded households (OR 1.35 [95% CI 1.15–1.58]). Pneumococcal carriage was detected less commonly among those who reported recent antibiotic use (OR 0.73 [95% CI 0.55–0.98]) and in *Staphylococcus aureus* carriers (OR 0.56 [95% CI 0.44–0.72]) than among those who were not (Table 3).

**Table 3:** Univariable and multivariable analysis of risk factors for carriage of any serotypeable pneumococcus among Aboriginal people in Western Australia, August 2008–November 2014

	Odds Ratio (95% Confidence Interval)	
	Any pneumococcal serotype	
	*Univariable*	*Multivariable*
Male	*1.56 (1.35*–*1.81)*	0.81 (0.67–0.99)
Age increase by 12 months		
0–11 months	*4.60 (2.83*–*7.50)*	*4.19 (2.39*–*7.33)*
1 to 19 yr	*0.85 (0.84*–*0.87)*	*0.85 (0.84*–*0.87)*
20 + years	0.99 (0.98–1.01)	1.00 (0.99–1.02)
Annual increase		
Pre-PCV13	1.07 (0.98–1.18)	*0.86 (0.75*–*0.98)*
Post-PCV13	0.88 (0.75–1.05)	*1.37 (1.08*–*1.74)*
Respiratory symptoms	*1.48 (1.14*–*1.94)*	1.01 (0.72–1.43)
Nasal discharge	*4.84 (4.01*–*5.74)*	*2.49 (2.00*–*3.09)*
Recent antibiotic use	*0.73 (0.55*–*0.98)*	*0.48 (0.33*–*0.69)*
Remoteness		
Metropolitan	Ref	Ref
Regional	1.36 (0.97–1.89)	1.31 (0.88–1.96)
Remote	*1.86 (1.47*–*2.35)*	*2.21 (1.67*–*2.92)*
ETS	*1.24 (1.06*–*1.46)*	1.06 (0.87–1.29)
≥5 people sharing (crowding)	*1.35 (1.15*–*1.58)*	*1.36 (1.11*–*1.67)*
Sharing with ≥1 child <5 years	1.15 (0.96–1.37)	1.07 (0.85–1.34)
Season		
Spring	Ref	Ref
Summer	0.91 (0.66–1.27)	0.95 (0.62–1.45)
Autumn	0.86 (0.70–1.07)	1.35 (0.91–2.00)
Winter	*1.29 (1.08*–*1.53)*	1.11 (0.73–1.69)
*H. influenzae*	*10.29 (8.60*–*12.30)*	N/A
*M. catarrhalis*	*7.10 (6.01*–*8.39)*	N/A
*S. aureus*	*0.56 (0.44*–*0.72)*	N/A

Entries in italic considered statistically significant results

In the multivariable analysis, significant independent predictors of carriage of any serotypeable pneumococcus included the presence of nasal discharge (OR 2.49 [95% CI 2.00–3.09]), crowding (OR 1.36 [95% CI 1.11–1.67]) and remote versus urban residence (OR 2.21 [95% CI 1.67–2.92]). Recent receipt of antibiotics was associated with lower odds of detection of pneumococcal carriage (OR 0.48 [95% CI 0.33–0.69]). After modelling for the influence of age using the spline function, there was a significant risk of carriage of pneumococcus up to age 1 year (OR 4.19 [95% CI 2.39–7.33]), which then decreased from 1 year to 19 years (14.7% decline per year; OR 0.85 [95% CI 12.9–16.4], Table 3). Adjusting for other factors, there was no evidence that introduction of PCV13 was associated with an overall reduction in all pneumococcal serotypes (Fig. 1).

## Discussion

The overall prevalence of carriage of pneumococci among WA Aboriginal children and adults remains high following the introduction of PCV13. For children, pneumococcal carriage has remained relatively stable over time, with carriage increasing in infants until around their first birthday and then slowly declining with increasing age to adulthood (Table 3).

IPD rates caused by non-PCV serotypes have increased since the introduction of PCV13 nationwide [4, 7]. In WA, the continuing high rates of carriage despite vaccination indicate an ongoing contribution of environmental factors to acquisition of pneumococcus.

The main factors associated with detection of pneumococcal carriage in our analysis were young age, presence of nasal discharge and household crowding. The age distribution of pneumococcal carriage—increasing each month of the first year and declining slowly thereafter—has been observed previously in Aboriginal children in WA [9] and correlates with the observed age distribution of OM which is hyper-endemic in this population [3, 16]. Nasal discharge was strongly associated with detection of pneumococcal carriage (Table 3). The authors speculate that pneumococcus plays a causative role in nasal discharge; however, it is also possible that nasal discharge merely improves detection of carriage. In either case, reinforcement of hygiene practices would be important for limiting transmission of pneumococcal strains.

The relationship between household crowding and pneumococcal carriage has been reaffirmed in this study. Previously crowding was linked with high carriage in a study conducted in communities surrounding Kalgoorlie in WA [17] and in other indigenous communities including Alaska Native people [18]. While not shown to be an independent risk factor in this analysis, ETS exposure was reported more frequently among those with pneumococcal carriage (univariable OR 1.24 [95% CI 1.06–1.46]) and this has previously been associated with both pneumococcal carriage [18] and OM in Aboriginal children [8, 19, 20]. These associations reinforce the need to promote healthy living conditions for Aboriginal people and the importance of smoking prevention programs.

Recent antibiotic use was strongly negatively associated with detection of pneumococcal carriage (Table 3). There was no appreciable change in rates of antibiotic resistance among carriage isolates over the study, apart from a decrease in resistance to cotrimoxazole in the post-PCV13 period (Table 2). Crude χ^2^ tests of carriage of non-susceptible strains of pneumococcus or multi-resistant strains versus recent antibiotic use identified no significant differences between the groups (data not shown). Taken together, these findings suggest that recent antibiotic use may be protective against pneumococcal carriage.

After adjusting for age, date, gender, remoteness, and other factors, there was no evidence that the prevalence of carriage of all serotypeable pneumococci across all age groups decreased after introduction of PCV13 (Table 3). Among children <5 years old, those most likely to have been vaccinated with PCV13, the prevalence of carriage across all serotypes did not change significantly (Table 1) but there was evidence of a significant decrease in carriage of both PCV7 and PCV13 serotypes (Table 1, Fig. 2). A similar reduction in vaccine serotypes was previously reported in Alaska Native children [21]. It appears that non-vaccine strains are replacing vaccine-type strains after PCV13 introduction, rather than PCV13 reducing carriage overall. This study was unable to investigate possible herd immunity in more detail, particularly in infants <6 months old, due to low enrolment numbers in this age group. Carriage of PCV13 serotypes was also numerically reduced in the older age groups studied (Table 1), albeit to a lesser extent than in children <5 years old. Post-PCV13 vaccination coverage was estimated at ≥82% among 5–14 year olds (Table 1), and the Australian National Centre for Immunisation Research and Surveillance estimates PCV vaccination coverage in Aboriginal children in WA at >88% [22]. The numerical reduction in PCV serotypes in older study participants is consistent with an indirect effect of PCV13.

There were a number of strengths to this study, chiefly the large sample size, spread over a broad geographic region over several years. However there were also several limitations. The opportunistic nature of sampling meant the study did not achieve similar coverage of inhabitants in all communities, and certain seasons were favoured for visiting certain areas due to easier access by road/air compared to rainy seasons. This meant some regions/seasons may have been comparatively under-represented in some analyses. Immunization status for all children in this study could not be ascertained, due to an inability to identify ACIR records for some participants. This was primarily due to the method used to access immunization data: participants were identified by name and date of birth, as documented on records during interviews. It is common among Aboriginal communities for individuals to be identified by several different names and spellings can vary for the same person; thus the data could not always be matched to a record on the ACIR database.

Because most children <5 years old post-PCV13 appeared to have received at least one dose of PCV13, the reduction in PCV13-non-PCV7 serotypes may have been directly influenced by PCV13 vaccination. However, greater decreases in PCV13 serotype carriage were noted in Alaskan [23] and Massachusetts children [24] following PCV13 introduction. Due to the uncertainty in vaccination status for a large proportion of participants, and the small number of children confirmed as PCV13 vaccinated (*n* = 37, Table 2), vaccination status was not included as a variable in the risk factor analysis. Another limitation of the study was the lack of detailed information to allow accurate assessment of indirect effects within households. It would be highly informative to analyse carriage and possible transmission between household members, and to assess the impact of vaccination status of children on other household members. However, in the communities studied, movement of family members between houses and communities is often dynamic rather than static, so such an analysis is likely to be imprecise.

Despite observing reduced carriage of 6C and 19A in children, these serotypes remained among the most common serotypes carried following PCV13 introduction. The reduction in carriage of serotypes 6A, 23F and 19A is plausibly attributable to PCV13 as they are vaccine serotypes. The reduction in 6C in children <5 years old may be attributable to cross-protection from 6A which is covered by PCV13 [25]. The increase in non-vaccine types, most notably 11A, 7B and 15B warrants further monitoring. Increases in 11A and serogroup 15 have been reported from other regions and countries. IPD caused by serogroup 15 increased in Norway after PCV13 introduction [26]. Serotype 11A was identified as an emerging serotype in the Northern Territory Aboriginal population following PCV7 introduction [27], and carriage of 11A also increased in Sweden and Japan following PCV13 introduction [28, 29]. Moreover, high rates of antimicrobial resistance among serotypes 23F, 7B and 9 V are particularly concerning and warrant close surveillance (Table 2).

## Conclusions

While PCV13 appears to have influenced the distribution of carriage serotypes among Aboriginal children in WA, the overall rate of pneumococcal carriage remains high across all age groups, with replacement of vaccine serotypes with emerging non-vaccine serotypes. The relevance of emerging carriage serotypes to IPD and more common pneumococcal manifestations of OM and pneumonia needs to be carefully assessed to properly understand the impact of conjugate vaccination.

## Abbreviations

23vPPV: 23-valent pneumococcal polysaccharide vaccine
ACIR: Australian Childhood Immunisation Register
CI: Confidence interval
ETS: Environmental tobacco smoke
IPD: Invasive pneumococcal disease
OM: Otitis media
OR: Odds ratio
PCV13: 13-valent pneumococcal conjugate vaccine
PCV7: 7-valent pneumococcal conjugate vaccine
WA: Western Australia
